# A Case of Unilateral Blaschko-Linear Lichen Planus Pigmentosus in a Seven-Year-Old Female: A Rare Presentation

**DOI:** 10.7759/cureus.41354

**Published:** 2023-07-04

**Authors:** Njoud AlNodali, Abdulellah I Aleissa

**Affiliations:** 1 Dermatology, King Fahad General Hospital, Jeddah, SAU; 2 Dermatology, King Abdulaziz University Faculty of Medicine, Jeddah, SAU

**Keywords:** pediatric skin, rare skin disease, blaschko lines, blaschko linear lichen planus pigmentosus, lichen planus pigmentosus

## Abstract

Lichen planus pigmentosus (LPP) is a rare form of lichen planus that typically affects middle-aged people with darker-pigmented skin. LPP is associated with a longer clinical course than classical lichen planus, which distinguishes it clinically. Its occurrence in children is uncommon, with few reported cases in this population in the literature. We report a rare presentation of unilateral blaschkoid LPP in a seven-year-old Saudi Arabian female patient.

## Introduction

Lichen planus pigmentosus (LPP) is an uncommon variant of lichen planus (LP) [1]. It is characterized by dark brown macules that are primarily found in the body's sun-exposed regions; its cause is unknown and it has an invasive and protracted course. While the disease may affect the mucosa, it does not affect the scalp or the nails [2]. In this case report, we present a seven-year-old female who presented with a history of left-thumb trauma followed by an asymptomatic purple papular skin rash. The rash had gradually spread to the dorsum of her left hand, before proceeding to appear over her left flank, eventually spreading to her left chest, left abdomen, and left back in a straight line. On examination, she had liveo-violaceous, S-shaped, and linear-patterned macules and papules in the pattern following Blaschko's lines. Neither the nails nor the mucosa were involved. This is a rare case of unilateral blaschkoid LPP. Only a few cases of this condition have been reported in the literature, and most of them involve adults.

## Case presentation

**Figure 1:**
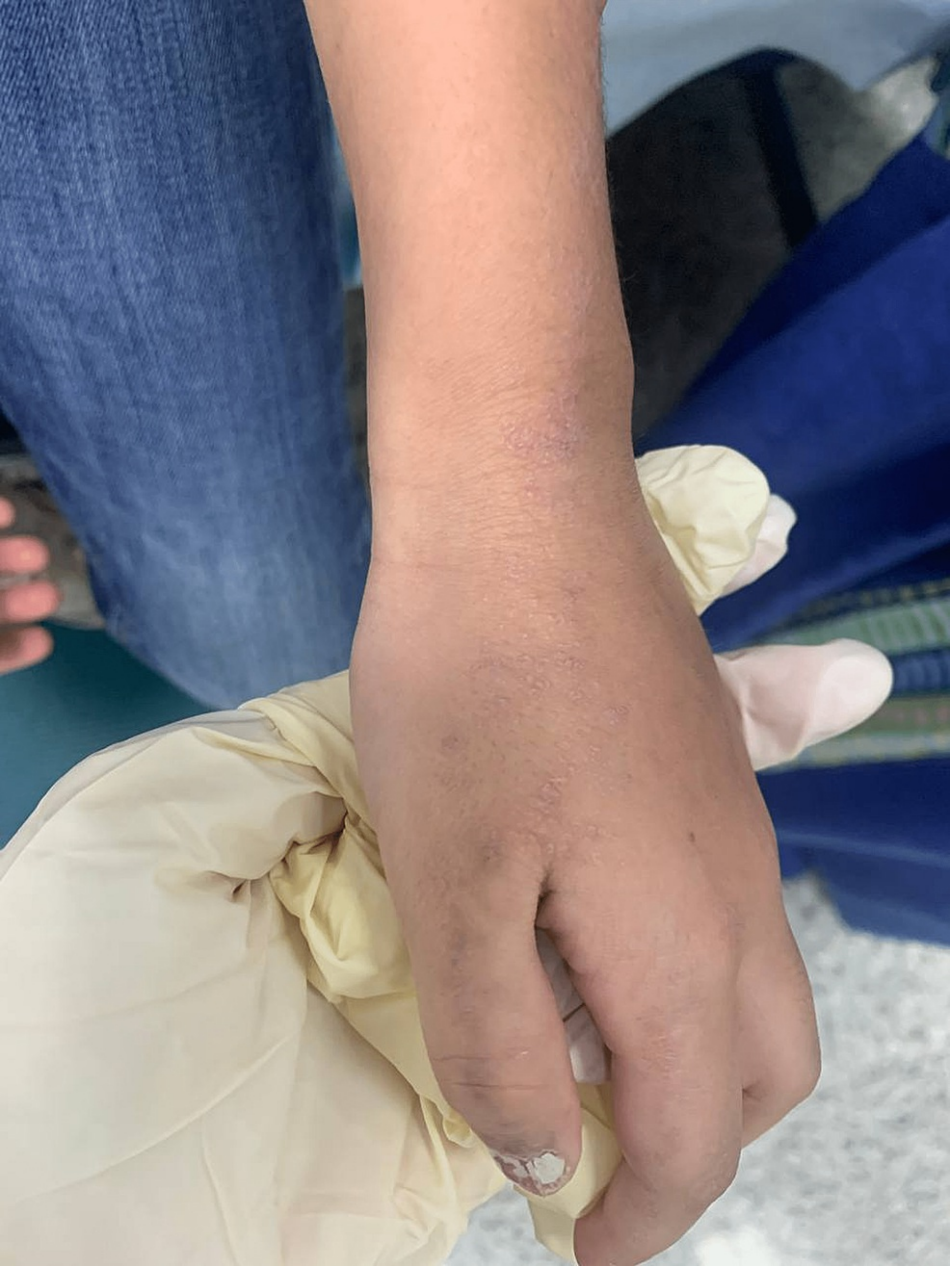
Left thumb showing purple papular skin rash

A medically free seven-year-old female presented with a history of left thumb trauma that had occurred three months ago, followed by the appearance of an asymptomatic purple papular skin rash over her left thumb (Figure 1).

**Figure 2:**
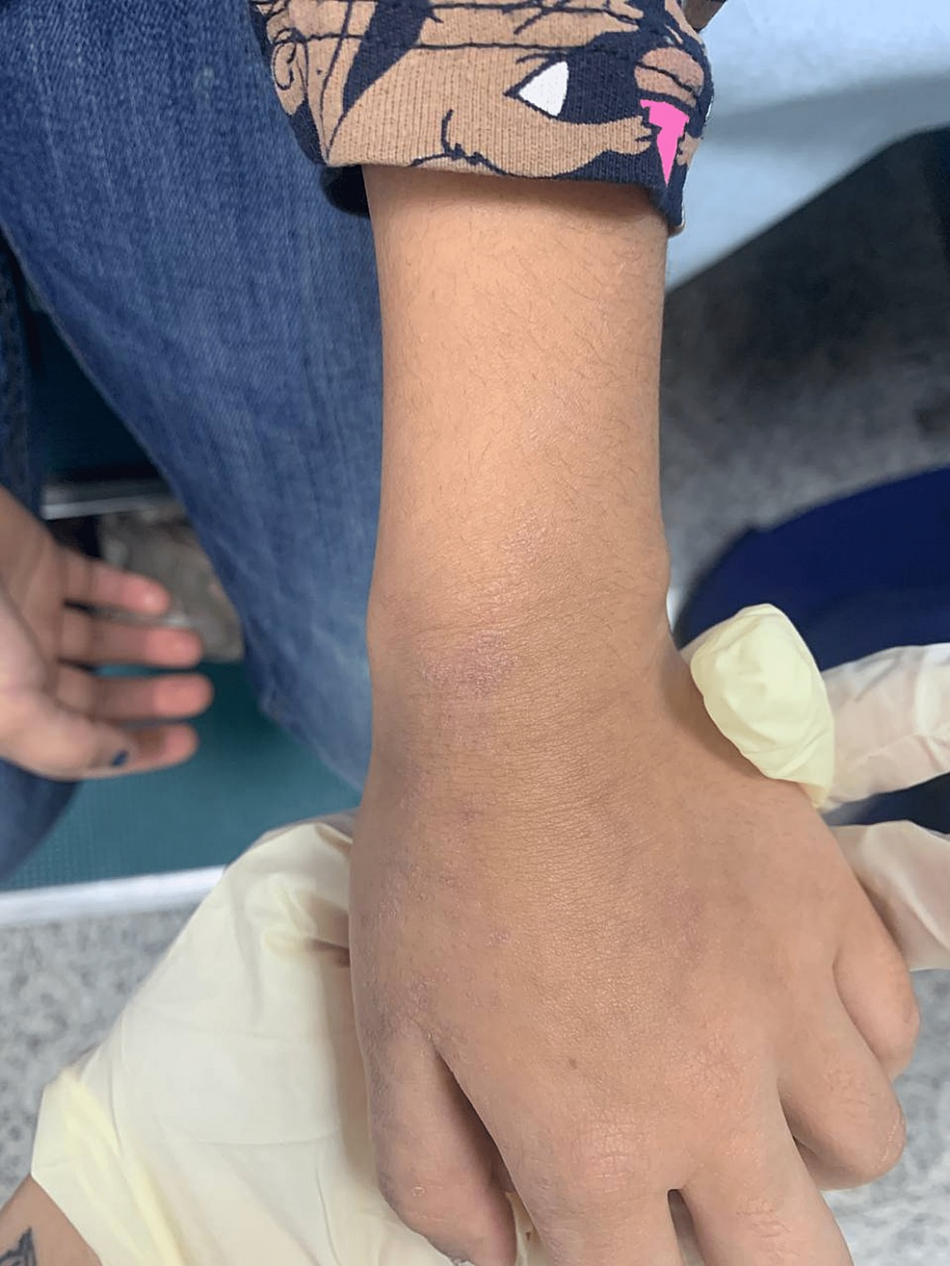
Dorsum of the left thumb showing skin rash

**Figure 3:**
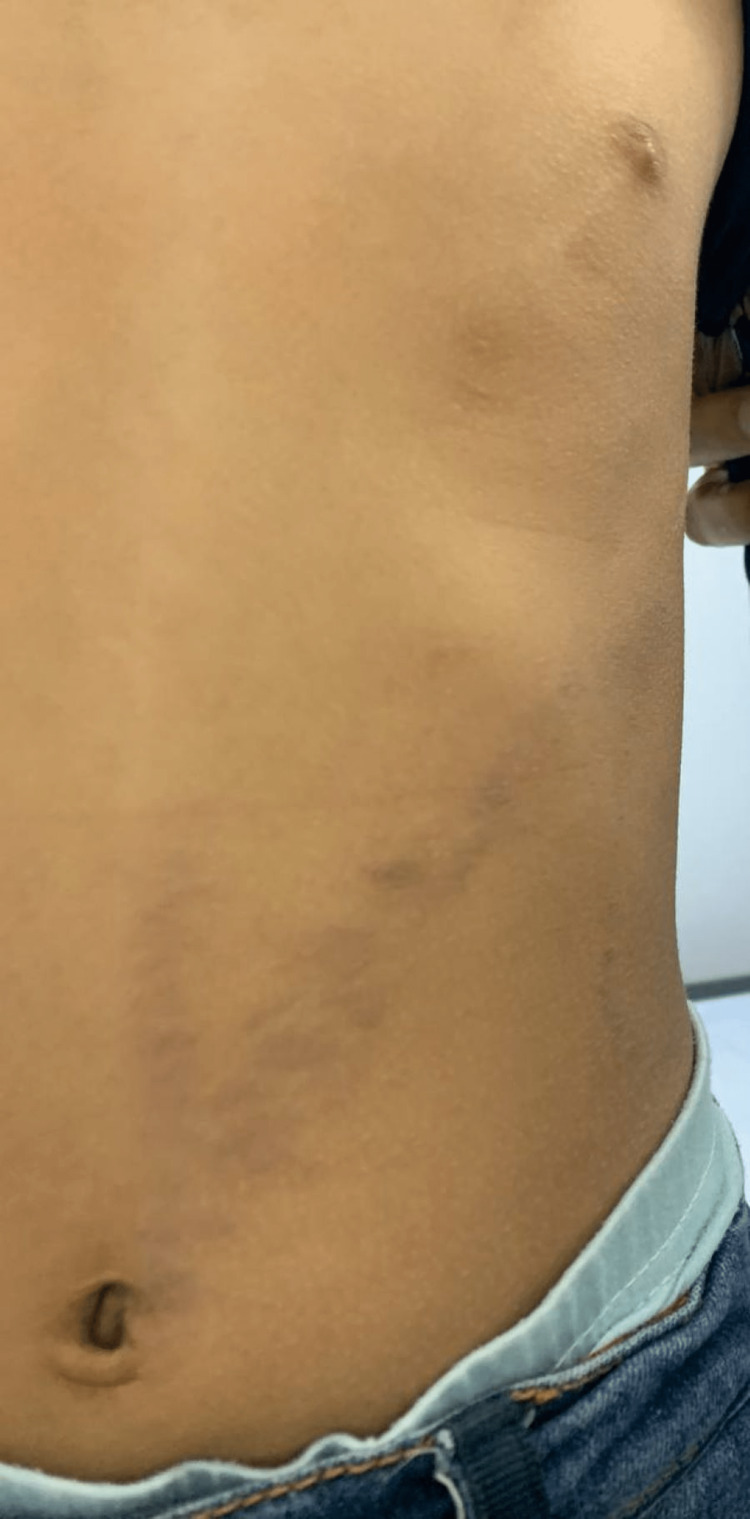
Left flank showing skin rash

**Figure 4:**
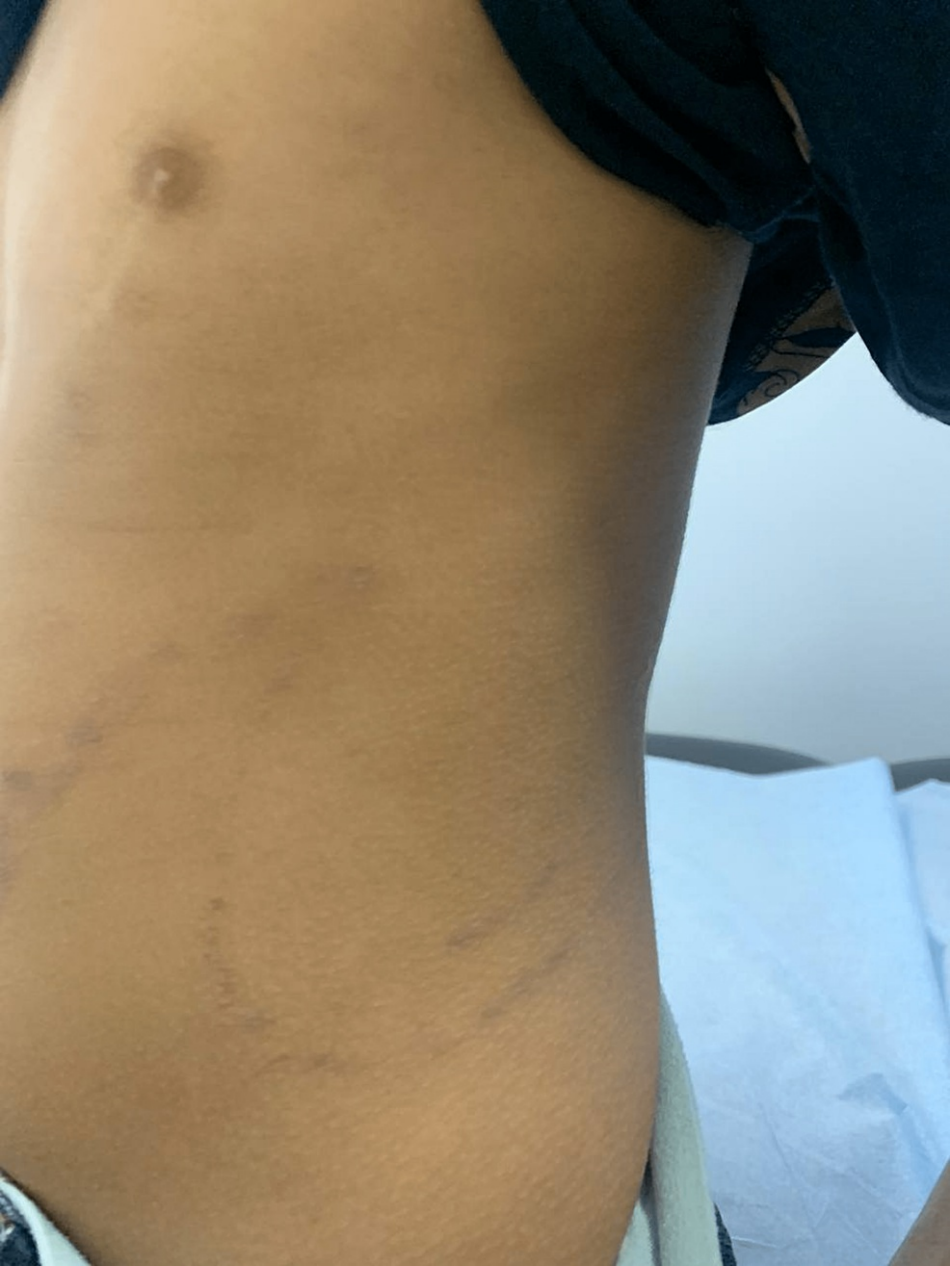
Left abdomen showing skin rash

**Figure 5:**
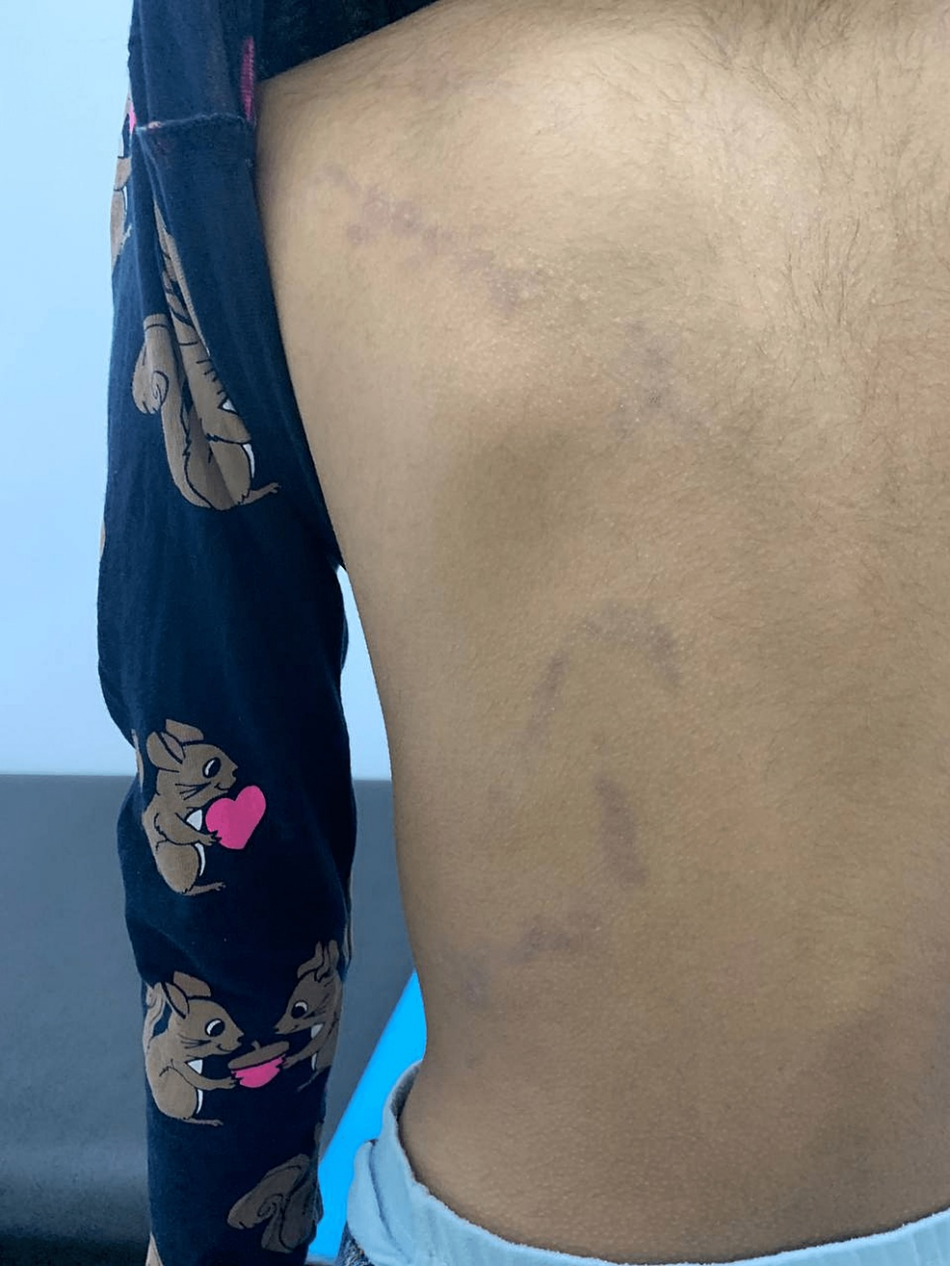
Rash on left back

A month after the initial trauma, she had started to develop the same skin rash over the dorsum of her left hand (Figure 2), which then proceeded to appear over her left flank, eventually spreading to her left chest (Figure 3), left abdomen (Figure 4), and left back (Figure 5) in straight lines.

The rash exhibited an unclear boundary and did not affect the right side of the body, ceasing precisely at the midline in both the anterior and posterior parts of her trunk. Apart from the thumb trauma, she had no history of drug intake, topical application, sun exposure, or contact with chemicals or plants. Liveo-violaceous, S-shaped, and linear-patterned macules and papules were observed during the dermatological examination, covering the right half of her abdomen and extending to the right lumbar region. There was also a tendency for coalescing macules and papules, which were observed extending from the left abdomen to the left lumbar area, stopping in the midline. There were no indications of involvement on the scalp, nails, or mucous membranes. Furthermore, there was no sign of lymphadenopathy. All her routine laboratory tests were within normal limits. The final diagnosis was unilateral blaschkoid LPP. The patient was given topical steroids, but she was lost to follow-up.

## Discussion

LPP usually manifests as mottled or reticulated areas of increased pigmentation, appearing as dark brown spots on the areas of the skin exposed to the sun. These macules can also appear on flexural folds on the face and neck, as well as the axillae and inguinal regions [3]. Basically, it affects adults after the age of 30 years [4]. Although it affects both sexes, it is more commonly found in females [5]. According to reports, it is more likely to occur in people with darker skin. Typically, the disease initially affects the face and neck before moving on to the upper extremities and trunk. Clinically, macules, popular lesions, and plaque-like lesions are frequent manifestations in LPP patients. Rare cases of the lesions becoming Koebnerized have also been reported. The lesions begin as small, ill-defined oval-to-round macules and evolve into confluent lesions affecting sizable areas [5]. LPP may manifest as a lichenoid reaction to a number of unidentified agents/incidents, including trauma, ultraviolet light, viral infections, and vaccinations, despite the fact that it is an idiopathic disease with no known cause [6,7].

According to the histopathological analysis, the condition is associated with hyperkeratosis, dermal atrophy with loss of rete pattern, focal basal cell vacuolization, and sparse dermal infiltrate [8]. The disease's histopathological manifestations include basal cell layer degeneration, melanin incontinence, moderate dermal fibrosis, epidermis atrophy, and the presence of Civatte bodies [5]. However, little is known about the etiopathology of LPP. The immunopathogenesis of LPP and LP is similar, with CD8+ T lymphocytes recognizing and attacking epidermal keratinocytes to cause keratinocyte degeneration [9] and severe pigmentary incontinence [10]. This altered cellular immune response is mediated by T lymphocytes.

The pigmentation may be perifollicular, diffuse, reticulate, or blotchy [4]. The distribution of the patches is typically symmetrical, but a few cases of LPP with segmental, blaschkoid, and zosteriform patterns have been documented in the literature [7,11]. There are not many cases of LPP with Blaschkoid distribution reported in the literature.

Bhutani et al. first described Blaschko's lines, which are lines of typical cell development [3]. Dossi Cataldo et al. have reported the case of a 48-year-old patient with three clinical subtypes [12], while Vineet et al. have described linear and zosteriform lesions that were lateralized to the right side of the body along the lines of Blaschko [13]. The fourth case of LPP was reported by Polat et al., on the trunk in the pattern that followed Blaschko's lines [14]. Additionally, mucosal or nail involvement is absent in the majority of the reported cases of LPP with Blaschkoid distribution, as seen in our case [7,12]. To the best of our knowledge, this is the first reported case of unilateral LPP with Blaschko’s line distribution in a pediatric patient in Saudi Arabia. The patient’s symptoms were similar to those in other reported cases of unilateral Blaschkoid LPP in the literature [12,15].

## Conclusions

To the best of our knowledge, this is the first reported case of unilateral Blaschko-linear LPP in a pediatric patient in Saudi Arabia. Due to the condition's rarity and ambiguous symptoms, it should be taken into account in the differential diagnosis of macular hyperpigmentation. The exact cause of the disease remains uncertain, emphasizing the need for further research into its etiology, pathogenesis, and treatment.
